# Urinary KIM-1 for Early Detection of Acute Kidney Injury in Neonates: A Systematic Review and Meta-Analysis

**DOI:** 10.3390/life15121842

**Published:** 2025-11-30

**Authors:** Manapat Praditaukrit, Moragot Chatatikun, Aman Tedasen, Suntornwit Praditaukrit, Sirihatai Konwai, Jason C. Huang, Wiyada Kwanhian Klangbud, Atthaphong Phongphithakchai

**Affiliations:** 1 Division of Neonatology, Department of Pediatrics, Faculty of Medicine, Prince of Songkla University, Songkhla 90110, Thailand; manapat.p@psu.ac.th; 2 School of Allied Health Sciences, Walailak University, Nakhon Si Thammarat 80160, Thailand; aman.te@wu.ac.th; 3 Research Excellence Center for Innovation and Health Products (RECIHP), Walailak University, Nakhon Si Thammarat 80160, Thailand; 4 Nephrology Unit, Division of Internal Medicine, Faculty of Medicine, Prince of Songkla University, Songkhla 90110, Thailand; suntornwir.p@psu.ac.th (S.P.); sirihataikonwai@gmail.com (S.K.); 5 Department of Biotechnology and Laboratory Science in Medicine, National Yang Ming Chiao Tung University, Taipei 112304, Taiwan; jasonhuang@nycu.edu.tw; 6 Medical Technology Program, Faculty of Science, Nakhon Phanom University, Nakhon Phanom 48000, Thailand; wiydakhanhian@gmail.com

**Keywords:** acute kidney injury, AKI, kidney injury molecule-1, KIM-1, neonates

## Abstract

Acute kidney injury (AKI) is a significant clinical concern in neonates, threatening optimal outcomes. Early and accurate diagnosis is crucial; however, current methods lack sufficient sensitivity. This meta-analysis aimed to evaluate urinary kidney injury molecule-1 (uKIM-1) for AKI in neonates by quantifying differences in uKIM-1 levels between AKI and non-AKI neonates. We systematically searched major databases for comparative studies. Quality assessment was performed using the Newcastle-Ottawa Scale, and the certainty of the evidence was assessed according to the Grading of Recommendations Assessment, Development and Evaluation (GRADE) methodology. A random-effects meta-analysis estimated the pooled Hedges’ g in uKIM-1 levels, accounting for heterogeneity. Subgroup analyses explored sources of heterogeneity (continent, study design, sampling time, AKI definition). Publication bias was assessed using Egger’s and Begg’s tests, as well as with a funnel plot. Data from 13 studies involving 552 neonates indicated a significant association between elevated uKIM-1 levels and AKI. High heterogeneity was observed (*I*^2^ = 80.32%). The pooled Hedges’ g was 0.62 (95% CI: 0.16–1.07, *p* = 0.01). Subgroup analysis showed stronger associations in African studies (Hedges’ g = 2.12), those using KDIGO (Hedges’ g = 0.96), cohort studies, and sampling within 2–4 days (Hedges’ g = 0.76). No publication bias was detected. This meta-analysis synthesizes evidence on uKIM-1 as an AKI biomarker. While uKIM-1 shows promise, high heterogeneity and diagnostic performance warrant further research to improve AKI detection and management in neonates.

## 1. Introduction

Acute kidney injury (AKI) represents a significant clinical challenge in neonates, associated with substantial morbidity, mortality, and long-term adverse outcomes [1,2]. Neonates are particularly vulnerable to AKI due to their immature renal physiology, limited compensatory reserve, and exposure to various stressors such as prematurity, asphyxia, and nephrotoxic medications [3,4,5]. Early diagnosis and appropriate management of AKI are crucial to mitigate its potential consequences; however, traditional diagnostic markers often lack the sensitivity and specificity required for timely intervention [6]. Therefore, there is a pressing need to identify and validate novel biomarkers that can facilitate early and accurate detection of AKI in neonates [7].

Historically, different classification systems have been used to define AKI, including the neonatal RIFLE (Risk, injury, failure, loss of kidney function, and end-stage kidney disease), AKIN (acute kidney injury network), and modified neonatal KDIGO (kidney disease: improving global outcomes) criteria [8,9,10]. While these criteria have improved AKI diagnosis, their application in neonates faces challenges [11]. They primarily rely on serum creatinine, which is influenced by maternal creatinine levels at birth and may not reflect true neonatal kidney function early in life [12]. Urine output can also be difficult to accurately measure in neonates [13]. These limitations highlight the need for more sensitive and specific biomarkers that can complement or even replace these traditional criteria, particularly in the neonatal population.

To address these limitations, recent research has focused on identifying more reliable biomarkers for early AKI diagnosis. To improve early detection, recent studies have explored both invasive and non-invasive biomarkers. Invasive markers like serum cystatin C, neutrophil gelatinase-associated lipocalin (NGAL), interleukin-18 (IL-18), and liver-type fatty acid-binding protein (L-FABP) offer enhanced sensitivity but require blood sampling, which is less ideal for neonates [14]. Non-invasive urinary biomarkers including NGAL, L-FABP, the combination of tissue inhibitor of metalloproteinases-2 (TIMP-2) with insulin-like growth factor-binding protein 7 (IGFBP7), and kidney injury molecule-1 (KIM-1) provide safer alternatives and reflect tubular injury or stress [15,16,17]. Additional promising markers such as uromodulin (UMOD) [18], apolipoprotein M (ApoM) [19], matrix metalloproteinase-7 (MMP-7) [20], and urinary albumin [21] have shown diagnostic value, while emerging candidates like galectin-3 [22], growth differentiation factor 15 (GDF-15) [23], monocyte chemoattractant protein-1 (MCP-1) [24], and miR-21a-5p [25] are under investigation for their roles in renal inflammation and injury.

Among these, urinary KIM-1 (uKIM-1) stands out due to its high specificity for proximal tubular damage. Unlike other markers influenced by systemic factors, KIM-1 is selectively expressed in injured tubular epithelial cells and absent in healthy kidneys [26]. Its urinary levels rise earlier than serum creatinine and correlate well with histopathological damage [27]. Recent advances, including uKIM-1-targeted nanoprobes and rapid detection platforms, further enhance its diagnostic utility, positioning it as a leading candidate for early, non-invasive AKI diagnosis in neonates [28]. Previous studies have explored the utility of uKIM-1 in various clinical conditions and populations. ElSadek et al., 2020 investigated uKIM-1 as a urinary biomarker of acute kidney injury in critically ill neonates, while Rumpel et al., 2022 assessed urine biomarkers, including uKIM-1, for the assessment of AKI in neonates with hypoxic–ischemic encephalopathy receiving therapeutic hypothermia [29,30]. However, the results have been variable, with some studies demonstrating significant differences in uKIM-1 levels between AKI and non-AKI groups, while others have reported more modest or non-significant findings [31,32].

Although uKIM-1 has shown promise as a biomarker for AKI, existing studies present inconsistent findings. A pediatric meta-analysis reported significantly elevated uKIM-1 levels in stage 2–3 AKI, but only modest increases in stage 1, with diagnostic value limited to early measurement windows [33]. In adults, uKIM-1 has demonstrated variable performance, with some studies reporting good sensitivity and specificity [34], while others found only moderate diagnostic accuracy, particularly in intensive care unit (ICU) settings [35]. These discrepancies highlight the need for a focused evaluation of uKIM-1 in neonates. To date, no meta-analysis has specifically assessed uKIM-1’s diagnostic performance in neonatal AKI. This systematic review and meta-analysis aimed to fill that gap by quantifying differences in uKIM-1 levels between AKI and non-AKI neonates, evaluating its diagnostic accuracy, and exploring sources of heterogeneity.

## 2. Materials and Methods

### 2.1. Protocol Registration

This systematic review and meta-analysis were prospectively registered in PROSPERO (Registration No. CRD420251144066; available at https://www.crd.york.ac.uk/PROSPERO/view/CRD420251144066 (accessed on 9 September 2025)) and conducted in accordance with the PRISMA 2020 guidelines (Preferred Reporting Items for Systematic Reviews and Meta-Analyses), as shown in Table S1. [36].

### 2.2. Search Strategy

To identify relevant studies, a systematic literature search was conducted across PubMed, Scopus, Embase (via Ovid), Web of Science, and the Cochrane Library. The search strategy, outlined in Tables S2–S6, was formulated to identify studies assessing uKIM-1 levels in neonates with and without AKI through relevant keywords. The search was limited to articles published in English, with no date restrictions, up to 17 September 2025.

### 2.3. Eligibility Criteria

Studies were selected based on predefined eligibility criteria. Inclusion criteria were: (1) participants were neonates, defined as infants aged 0 to 28 days inclusive, encompassing both preterm and full-term infants; (2) participants were required to have either a diagnosis of AKI based on established criteria, such as modified neonatal kidney disease: improving global outcomes (nKDIGO), acute kidney injury network (AKIN), or neonatal risk, injury, failure, loss of kidney function, and end-stage kidney disease (nRIFLE), or to belong to a distinct comparator group of neonates without AKI; (3) eligible study designs included randomized controlled trials (RCTs) and observational studies, specifically cohort studies (prospective or retrospective) and case–control studies. Both cohort and case–control (including nested case–control) studies were included as pre-specified in the PROSPERO protocol because neonatal AKI biomarker data remain limited, and several key uKIM-1 publications used these designs. All case–control studies included clearly defined non-AKI comparators and standardized AKI definitions, enabling consistent effect-size estimation across designs; and (4) studies that were available as full-text articles in English.”

Exclusion criteria were: studies involving participants outside the specified neonatal age range; studies that did not provide separate data for the neonatal subgroup; studies lacking a clear, predefined, and internationally recognized definition of AKI; reviews (systematic reviews, meta-analyses, literature reviews), editorials, commentaries, letters to the editor, expert opinions, guidelines, position statements (unless they included original data), and conference abstracts; and studies not available as full-text articles in English.

### 2.4. Study Selection

M.P. and M.C. independently screened titles and abstracts to assess eligibility based on the predefined inclusion and exclusion criteria. Interrater reliability for both the title-abstract and full-text screening phases was assessed using Cohen’s Kappa (κ) [37]. The kappa coefficient was interpreted on the following scale: 0–0.20 (No agreement), 0.21–0.39 (minimal agreement), 0.40–0.59 (weak agreement), 0.60–0.79 (moderate agreement), 0.80–0.90 (strong agreement), and above 0.90 (almost perfect agreement). Subsequently, full-text articles of potentially relevant studies were retrieved and assessed against those criteria. Any disagreements between the reviewers were resolved through discussion or, when necessary, by consulting a third reviewer, A.P.

### 2.5. Data Extraction

Data from eligible studies were independently extracted by two reviewers (M.P. and M.C.) using a standardized data extraction form. The following information was collected: study characteristics (author, year conducted, study design), participant description, gestational age, sampling time (days), setting, AKI definition, number of AKI and non-AKI participants, percentage of male participants, and assay method.

After extraction, the reviewers systematically compared each data item in the form against the original article. Any discrepancies were first discussed to determine whether they resulted from interpretation differences, transcription errors, or unclear reporting in the original article. If agreement could not be reached through discussion, a third reviewer (A.P.) adjudicated the decision. When disagreements were due to incomplete or ambiguous data in the primary study, the corresponding authors were contacted for clarification. All decisions were documented to maintain transparency and reproducibility. Authors of primary studies were contacted to obtain missing or unclear information when possible.

### 2.6. Quality Assessment and Certainty of Evidence Assessment

The methodological quality of included studies was assessed independently by two reviewers (M.P. and M.C.) using the Newcastle-Ottawa Scale (NOS) [38]. This scale, adapted for both case–control and cohort study designs, evaluates study quality across three domains: selection (maximum 4 stars), comparability of study groups (maximum 2 stars), and exposure/outcome (maximum 3 stars). Each study was assigned a summary score based on the NOS criteria, with higher scores indicating higher methodological quality; total possible scores range from 0 to 9 stars. Studies scoring 7–9 stars were considered to be of high quality, 5–6 stars as moderate quality, and < 5 stars as low quality. Disagreements in quality assessment were resolved through discussion or, when necessary, consultation with a third reviewer (A.P.).

The certainty of evidence for the primary outcome (uKIM-1 levels) was evaluated using the GRADE (Grading of Recommendations Assessment, Development and Evaluation) approach [39]. Evidence from observational studies commenced with an initial rating of low certainty. The certainty was then systematically evaluated across five domains for potential downgrades: risk of bias (assessed using the NOS for included studies), inconsistency (heterogeneity and variability of effects), indirectness (PICO mismatch), imprecision (confidence interval width and sample size), and publication bias (funnel plot symmetry and statistical tests).

### 2.7. Data Synthesis and Analysis

Data extracted included sample size (*n*), mean uKIM-1 levels (ng/mL) or ng/mg, and standard deviation (SD). When uKIM-1 levels were reported as medians with interquartile ranges (IQRs), these values were converted to estimates of the mean and standard deviation (SD) using the formulas described by Luo et al., 2018, with the mean approximated as (Q1 + Median + Q3)/3 and the SD as (Q3 − Q1)/1.35, assuming a normal distribution [40]. Prior to analysis, uKIM-1 values reported in pg/mL were converted to ng/mL using appropriate conversion factors. If a study reports the mean in pg/mL, divide the mean by 1000 to convert it to ng/mL. To convert a mean with a 95% confidence interval (CI) to the SD, the SD was estimated by subtracting the lower limit from the upper limit of the CI, dividing the result by 3.92, and then multiplying by the square root of the sample size (*n*) [41]. The median, along with the minimum and maximum values, was used to estimate the mean by adding the minimum value to twice the median, then dividing the total by four, and the standard deviation was approximated by dividing the difference between the maximum and minimum values by four, assuming a symmetric data distribution [42].

A random-effects meta-analysis, utilizing the DerSimonian-Laird method, was conducted to estimate the pooled Hedges’ g in uKIM-1 levels between neonates with (AKI group) and without AKI (non-AKI group). When units of measurement varied across studies, Hedge’s g correction was applied to adjust for small sample bias and ensure comparability of effect sizes. Heterogeneity among studies was evaluated using Cochran’s Q test and the *I*^2^ statistic to determine the level of variability in effect estimates. *I*^2^ values were interpreted as follows: 25% indicating low heterogeneity, 50% indicating moderate heterogeneity, and 75% indicating high heterogeneity [43].

To investigate heterogeneity, subgroup analyses were conducted based on continent, study design, sampling time, and AKI definition. Subgroup-specific effect sizes were calculated, and differences between subgroups were assessed using subgroup tests. To assess the robustness of results, a leave-one-out sensitivity analysis was performed by iteratively excluding each study and recalculating the pooled effect estimate [44]. Publication bias was assessed visually with funnel plot and statistically with Egger’s and Begger’s tests, with a *p*-value < 0.05 considered indicative of significant publication bias. All analyses were performed using Stata version 19 (StataCorp LLC, College Station, TX, USA).

## 3. Results

### 3.1. Search Results

**Figure 1:**
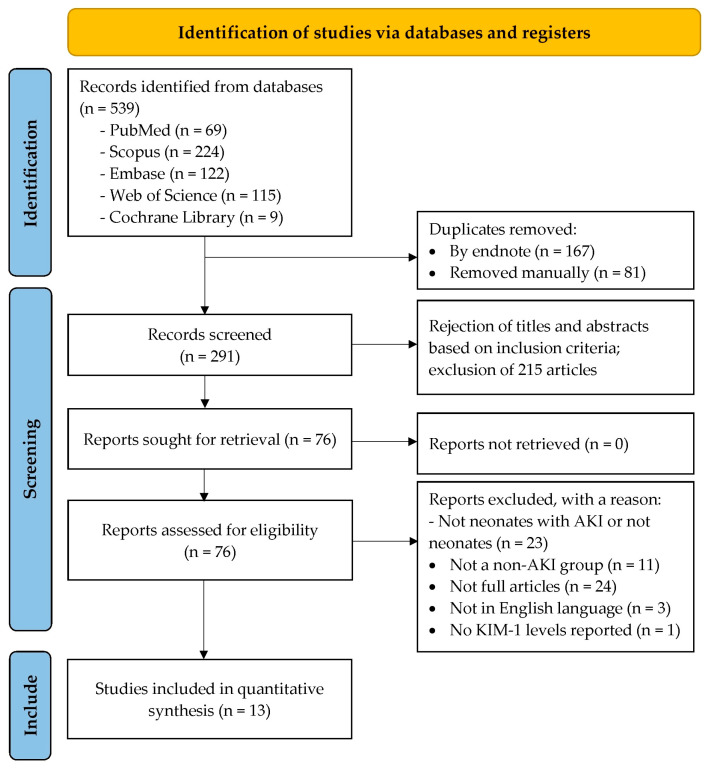
PRISMA flow diagram of study selection.

A total of 539 records were identified through database searching: PubMed (*n* = 69), Scopus (*n* = 224), Embase (*n* = 122), Web of Science (*n* = 115), and Cochrane Library (*n* = 9). After removing duplicates (*n* = 167 via EndNote; *n* = 81 manually), 291 records were screened based on titles and abstracts, leading to the exclusion of 215 articles. Seventy-six reports were sought for retrieval, all of which were successfully retrieved (*n* = 0 not retrieved). Following assessment for eligibility, 63 reports were excluded, for the following reasons: not neonates with AKI or not neonates (*n* = 23), not a non-AKI group (*n* = 11), not full articles (*n* = 24), not in English language (*n* = 3), and no uKIM-1 levels reported (*n* = 1). Ultimately, 13 studies were included in the quantitative synthesis (meta-analysis), as shown in Figure 1 [29,30,45,46,47,48,49,50,51,52,53,54,55]. Interrater reliability, assessed using Cohen’s Kappa, indicated almost perfect agreement for both title-abstract screening (κ = 0.87) and full-text screening (κ = 0.90), as shown in Tables S7 and S8.

### 3.2. Study Characteristics

The study characteristics were compiled from thirteen studies conducted across multiple countries: the Republic of Korea (*n* = 1) [45], the United States of America (USA) (*n* = 5) [29,46,47,48,54], Chile (*n* = 1) [49], Turkey (*n* = 3) [50,51,55], Egypt (*n* = 1) [30], Iran (*n* = 1) [52], and Greece (*n* = 1) [53] as shown in Table 1. The included studies spanned multiple continents, including Asia (*n* = 5) [45,50,51,52,55], North America [29,46,47,48,54] (*n* = 5), South America (*n* = 1) [49], Africa (*n* = 1) [30], and Europe (*n* = 1) [53]. Gestational ages ranged from extremely preterm to full-term infants [29,30,45,46,47,48,49,50,51,52,53,54,55]. The sampling times among the included studies primarily focused on the early postnatal period, with urine samples collected within the first 7 days of life in several studies [29,45,46,47,48,50,51,52,55]. Some samples were taken as early as 8 to 24 h post-bypass [16], or after anesthesia induction [49], or 3 days after admission [46], while others were extended up to 10 days [53]. The included studies employed the following study designs: cohort (*n* = 7) [29,45,48,49,51,54,55] and case–control studies (*n* = 6) [30,46,47,50,52,53]. Conditions studied were: premature infants [45,48], very low birth weight infants [46,48,55], neonates with birth weight > 2000 g [47], those with complex congenital heart diseases [49], non-septic and non-asphyxiated critically ill neonates [50], critically ill neonates [30], neonates with respiratory distress syndrome (RDS) [51], asphyxia [52,53], those undergoing hypoxic–ischemic encephalopathy receiving therapeutic hypothermia [29], and cardiac surgery (CS) [54]. The neonates’ ages ranged from postnatal day 1 to 23 days [29,30,45,46,47,48,49,50,51,52,53,54,55]. In studies where reported, the percentage of males in the AKI and non-AKI groups ranged from 36% to 89% and 38% to 80%, respectively [29,30,45,46,47,48,49,50,51,52,53,54,55]. All studies evaluated neonates within a neonatal intensive care unit (NICU) setting [29,30,45,46,47,48,49,50,51,52,53,54,55]. The AKI definitions used were nKDIGO (*n* = 8) [29,30,45,48,51,53,54,55], AKIN (*n* = 3) [46,47,50], and nRIFLE (*n* = 2) [49,52]. Across all studies, the total number of neonates was 552, with 192 diagnosed with AKI and 360 without AKI [29,30,45,46,47,48,49,50,51,52,53,54,55]. Biomarker measurements were analyzed using ELISA [29,30,45,46,47,48,49,50,51,52,53,54,55]. The uKIM-1 levels were reported as mean ± SD [29,30,45,51,52], median (IQR) [46,48,49,53,54], median (minimum-maximum) [50] and mean (95% CI) [47]. Measurements were reported as both absolute concentrations (pg/mL or ng/mL) [29,30,46,47,48,49,50,52,53,54,55] and as ratios normalized to urine creatinine (ng/mg) [45,51].

### 3.3. Results of Quality Assessment and GRADE Assessment of Evidence

Based on the Newcastle-Ottawa Scale (NOS) assessment of studies evaluating biomarkers for AKI in neonates, the majority of included studies were classified as high quality, as shown in Table 2 and Table S9. Eleven out of the thirteen studies received total scores ranging from 7 to 8 out of 9, indicating strong methodological rigor in terms of selection, comparability, and exposure or outcome assessment [29,30,45,46,47,48,50,52,53,54,55]. In contrast, studies by Borchet et al., 2021, and Genc et al., 2012 were rated as moderate quality, with scores of 6, primarily due to limitations in selection and comparability criteria [49,51]. Overall, the evidence base is robust, with most studies demonstrating high methodological quality, supporting the reliability of findings related to AKI biomarkers in neonatal populations.

**Figure 2:**
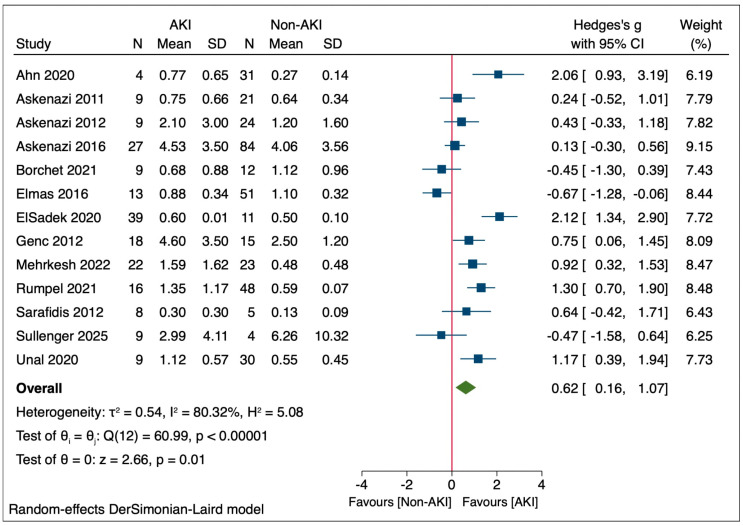
Forest plot of urinary kidney injury molecule-1 (uKIM-1) levels in neonates with and without acute kidney injury (AKI) [29,30,45,46,47,48,49,50,51,52,53,54,55]. Each blue square represents Hedges’s g, and its size reflects the study weight, while horizontal lines indicate the 95% confidence interval (CI). The green diamond represents the pooled effect size derived from a random-effects DerSimonian–Laird model. AKI, neonates with acute kidney injury; non-AKI, neonates without acute kidney injury.

The certainty of evidence for the association between uKIM-1 levels and AKI in neonates was assessed using the GRADE approach, as shown in Table S10. Based on 13 observational studies (192 AKI and 360 non-AKI neonates), the pooled effect size was Hedges’ g = 0.62 (95% CI: 0.16–1.07) as shown in Figure 2. The certainty was rated as low due to downgrades for inconsistency and imprecision. Inconsistency was considered serious because of substantial heterogeneity (*I*^2^ = 80.32%) despite subgroup analyses, resulting in two levels of downgrade. Imprecision was also serious due to the wide confidence interval and relatively small sample size, leading to one level of downgrade. Risk of bias, indirectness, and publication bias were not downgraded.

### 3.4. uKIM-1 Levels in Neonatal AKI

In this meta-analysis of 13 studies evaluating uKIM-1 in neonatal AKI, the pooled analysis demonstrated that the uKIM-1 levels were significantly higher in neonates with AKI compared to those without AKI (non-AKI), as shown in Figure 2 [29,30,45,46,47,48,49,50,51,52,53,54,55]. The overall standardized Hedges’ g was 0.62, with a 95% CI of 0.16 to 1.07, indicating a statistically significant association between elevated uKIM-1 and the presence of AKI (*p* = 0.01). The analysis was performed using a random-effect model (DerSimonian-Laird) to account for between-study variability. High heterogeneity was observed across studies (*I*^2^ = 80.32%, *p* < 0.00001), suggesting differences among the studies.

### 3.5. Subgroup Analysis

Subgroup analysis demonstrated significant differences in uKIM-1 levels between neonates with and without AKI across various subgroups, as shown in Table 3. When stratified by continent (test of group difference, *p* < 0.0001), the most pronounced effect was observed in Africa (Hedges’ g = 2.12, 95% CI: 1.34 to 2.90, *p* < 0.0001), although this was based on a single study, as shown in Table 3 and Figure S1 [30]. Asia showed a moderate effect size (Hedges’ g = 0.79, 95% CI: −0.05 to 1.62, *p* = 0.065) with high heterogeneity (*I*^2^ = 84.81%) [45,50,51,52,55], while Europe [53], North America [29,46,47,48,53], and South America [49] did not show statistically significant differences. In terms of study design (test of group difference, *p* = 0.95), cohort studies showed a statistically significant difference (Hedges’s g = 0.63, 95% CI: 0.02 to 1.23, *p* = 0.038, *I*^2^ = 78.54%) [29,45,48,49,51,54,55], while case–control studies did not reach statistical significance (*p* = 0.128), as shown in Table 3 and Figure S2 [30,46,47,50,52,53]. The test of group difference for sampling time showed no significant difference overall (*p* = 0.860), although samples collected within the first 2–4 days of life showed a significant difference (Hedge’s g = 0.76, *p* = 0.002; *I*^2^ = 68.21%), with moderate heterogeneity, as shown in Table 3 and Figure S3 [29,47,48,52,55]. In contrast, samples collected later (6–10 days) [45,46,50,51,53] or after induction of anesthesia at 24 h [49], 3 days after admission [30], or 8 to 24 h after separation from bypass [54] showed less consistent and non-significant findings (*p* = 0.208 and *p* = 0.650, respectively). Regarding AKI definitions (test of group difference, *p* = 0.10), studies using nKDIGO criteria revealed a significant elevation in uKIM-1 levels among AKI neonates (Hedges’ g = 0.96, 95% CI: 0.38 to 1.54, *p* = 0.001; *I*^2^ = 79.21%), indicating high heterogeneity [29,30,45,48,51,53,54,55], whereas AKIN and nRIFLE-based studies did not demonstrate significant differences, as shown in Table 3 and Figure S4 [46,47,49,50,52]. These findings suggest that the observed differences in uKIM-1 levels may be influenced by geographic region, diagnostic criteria, sampling time, and study design.

### 3.6. Sensitivity Analysis

**Figure 3:**
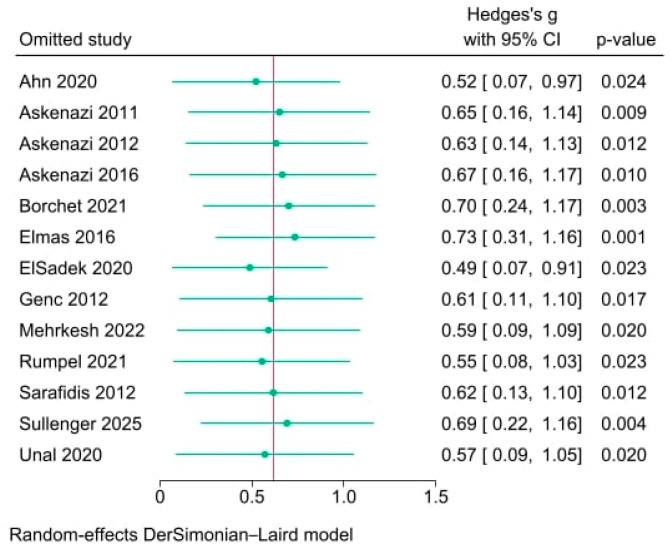
Leave-one-out sensitivity analysis demonstrating the effect of omitting individual studies on the pooled Hedges’ g in urinary kidney injury molecule-1 (uKIM-1) levels between neonates with and without acute kidney injury (AKI) [29,30,45,46,47,48,49,50,51,52,53,54,55]. The omitted Study identifies each study removed from the meta-analysis. Hedges’ g with 95% CI indicates the effect size and its uncertainty after omitting each study. The *p*-value represents the statistical significance of Hedges’ g after omitting the study. Horizontal lines display 95% confidence intervals for Hedges’ g. The vertical line shows the overall pooled Hedges’ g. Circles mark the point estimate of Hedges’s g after each omission. The analysis was performed using a random-effects DerSimonian-Laird model.

The leave-one-out sensitivity analysis demonstrated that the overall pooled Hedges’ g of uKIM-1 levels remained stable, regardless of which individual study was omitted. The pooled Hedges’ g values consistently ranged from 0.49 to 0.73, with all *p*-values remaining significant, indicating robust results as shown in Figure 3. None of the studies, when omitted, shifted the overall effect size outside the initial 95% CI, confirming that the meta-analysis findings were not unduly influenced by any single study. This underscores the reliability and stability of the overall conclusions regarding uKIM-1 levels in neonates with AKI.

### 3.7. Publication Bias

**Figure 4:**
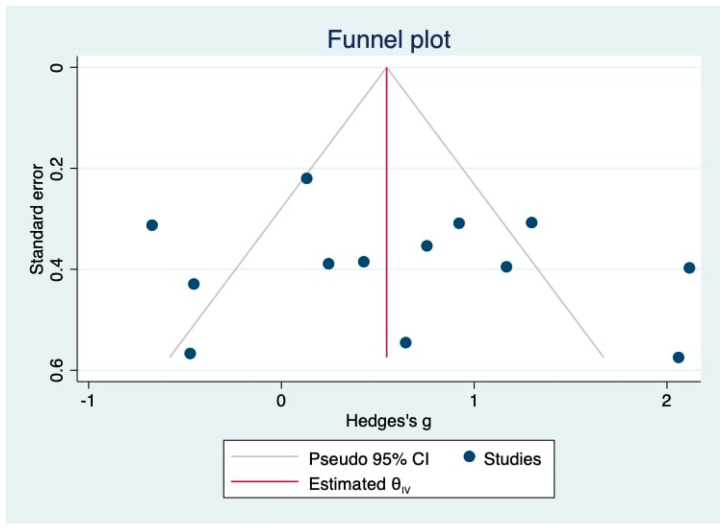
Funnel plot assessing publication bias for studies on urinary kidney injury molecule-1 (uKIM-1) levels in neonatal acute kidney injury (AKI) [29,30,45,46,47,48,49,50,51,52,53,54,55]. The funnel plot displays Hedges’ g plotted against the standard error of each study. The vertical red line represents the pooled effect size, while the pseudo 95% confidence limits are indicated by the diagonal lines. The relatively symmetrical distribution of studies around the pooled effect size suggests no apparent publication bias, which is further supported by Egger’s test (*p* = 0.6362) and Begg’s test (*p* = 0.7603).

Although Egger’s and Begg’s tests did not indicate significant publication bias (Egger’s test: *p* = 0.6362; Begg’s test: *p* = 0.7603), the interpretation should consider the limited number of studies (*n* = 13), which reduces the power of these tests [29,30,45,46,47,48,49,50,51,52,53,54,55]. Visual inspection of the funnel plot (Figure 4) revealed a relatively symmetrical distribution of effect sizes around the pooled estimate, suggesting minimal small-study effects. However, a slight dispersion among smaller studies was observed, particularly those with higher effect sizes, which may reflect heterogeneity rather than true bias. This qualitative assessment supports the statistical findings but underscores the need for cautious interpretation given the small sample size.

## 4. Discussion

This systematic review and meta-analysis synthesized data from 13 studies involving a total of 552 neonates to evaluate the association between uKIM-1 levels and AKI. The comprehensive search process identified a total of 539 records, with 13 studies ultimately meeting the inclusion criteria after rigorous screening. These studies spanned multiple countries, including the USA (North America), Turkey (Asia), Chile (South America), Egypt (Africa), Greece (Europe), Iran (Asia), and Korea (Asia), and involved diverse neonatal populations ranging from extremely preterm infants to full-term neonates. The inclusion of various study designs, predominantly cohort and case–control, provided a broad perspective on the potential utility of uKIM-1 as a biomarker for neonatal AKI. Despite most included studies demonstrating high methodological quality (high quality via NOS), the GRADE assessment assigned a low certainty to the observed association between uKIM-1 levels and AKI in neonates. This low rating was primarily driven by serious inconsistency (attributed to high heterogeneity) and imprecision (due to a wide confidence interval and relatively small sample size), although no significant publication bias, including small study effects via funnel plot symmetry, was detected. Differences between continents may reflect variation in population risk profiles, nephrotoxin exposure, and laboratory assays, while variation in sampling time is consistent with expected biomarker kinetics during early tubular injury. An exploratory meta-regression using gestational age and postnatal day at urine collection did not appreciably reduce heterogeneity, suggesting that residual clinical variability remains.

The meta-analysis revealed that uKIM-1 levels were significantly higher in neonates with AKI compared to those without, with a Hedge’s g of 0.62 (95% CI: 0.16 to 1.07, *p* = 0.01). Despite high heterogeneity among the included studies (*I*^2^ = 80.32%), the random-effects model accounted for this variability, indicating a consistent association across different populations. This supports the utility of uKIM-1 as a sensitive biomarker for early detection of AKI in neonatal populations. The result is consistent with previous studies included in the review, such as those by Askenazi et al., 2011, Askenazi et al., 2012 [46,47], ElSadek et al., 2020 [30], and Rumpel et al., 2021 [29], which demonstrated elevated uKIM-1 levels in neonates with AKI across various clinical conditions. Beyond the included studies, additional research further supports these findings. Lu et al., 2019 reported that uKIM-1 and NGAL levels were significantly elevated in preterm infants with AKI within the first three days of life, preceding changes in serum creatinine [31]. Similarly, Groves et al., 2022 demonstrated in a neonatal rat model of hypoxic–ischemic encephalopathy that uKIM-1 levels increased significantly following AKI, correlating with histological evidence of proximal tubular injury [56]. Moreover, similar meta-analyses in adult populations have reported increased uKIM-1 levels in AKI patients, supporting its broader applicability as a renal injury marker [34,57,58]. The consistency of these findings across multiple studies and age groups strengthens the evidence for uKIM-1 as a promising early diagnostic biomarker for AKI in neonates, potentially facilitating timely intervention and improved outcomes.

Subgroup analyses revealed important insights into the variability of uKIM-1 levels across different study characteristics. Geographic differences were also observed, with the strongest effect size reported in the African subgroup (Hedges’ g = 2.12), although this was based on a single study. Conversely, studies from North America and Europe showed smaller or non-significant differences, which may reflect regional differences in clinical practices, environmental factors, or patient characteristics [3,59]. Notably, studies using the nKDIGO criteria showed a significant association between elevated uKIM-1 levels and AKI, while those using AKIN and nRIFLE definitions did not reach statistical significance. The nKDIGO criteria may be more sensitive in neonates because they incorporate smaller relative creatinine changes and standardized urine-output thresholds, which better capture early and subtle tubular dysfunction. This supports the growing consensus that KDIGO offers improved sensitivity and clinical relevance in neonatal AKI diagnosis [60,61]. Furthermore, study design appeared to influence outcomes; cohort studies generally reported larger effect sizes, whereas case–control studies displayed more variability. These differences align with previous research indicating that heterogeneity in study populations and methodologies significantly affects biomarker performance and diagnostic accuracy [62,63].

When comparing uKIM-1 to other urinary biomarkers of AKI, such as neutrophil gelatinase-associated lipocalin (NGAL), cystatin C, and interleukin-18 (IL-18), uKIM-1 has shown promising specificity for tubular injury. NGAL is widely studied and often rises earlier than serum creatinine, but it may be influenced by systemic inflammation and sepsis, limiting its specificity in critically ill neonates [64,65]. Cystatin C, while useful in serum, has limited utility in urine due to variable filtration and reabsorption dynamics. IL-18 has been associated with ischemic injury, but lacks consistency across neonatal populations [66,67]. In contrast, uKIM-1 is upregulated specifically in proximal tubular epithelial cells following injury, making it a more direct marker of renal epithelial damage. Studies such as those by Askenazi et al., 2016 [48] and Sarafidis et al., 2012 [53] have compared multiple biomarkers and found uKIM-1 to be among the most reliable for distinguishing AKI from non-AKI states in neonates. However, combining biomarkers may enhance diagnostic accuracy, as demonstrated in multi-marker panels that integrate uKIM-1 with NGAL and IL-18 [3].

Sensitivity analysis confirmed the robustness of the pooled findings, demonstrating that omission of individual studies did not substantially alter the overall effect size or significance. The effect sizes remained within a narrow range, and all *p*-values continued to indicate significance, reinforcing the stability of the results. Egger’s and Begg’s tests indicated no publication bias; however, the limited number of studies reduces statistical power. The funnel plot appeared largely symmetrical with minor small-study dispersion, suggesting heterogeneity rather than true bias, which supports the overall validity of the meta-analysis but warrants cautious interpretation.

While these findings endorse uKIM-1 as a promising biomarker for neonatal AKI, several considerations remain. The high heterogeneity underscores the influence of factors such as geographic region, diagnostic criteria, and study design. Future research should aim for standardized AKI definitions and uniform biomarker measurement protocols to improve comparability. Larger, multicenter prospective studies are needed to validate uKIM-1’s diagnostic accuracy and prognostic value across diverse neonatal populations. Moreover, longitudinal studies could elucidate the temporal dynamics of uKIM-1 elevation relative to clinical AKI diagnosis, further establishing its clinical utility. Although standardized cut-off values of uKIM-1 are not yet established, several neonatal studies report moderate diagnostic accuracy with Area Under the Curve (AUC) values ranging from approximately 0.63 to 0.81 [51,55]. Combining uKIM-1 with complementary biomarkers such as NGAL may further improve diagnostic performance, and these approaches warrant future evaluation.

Several limitations should be acknowledged. First, all included studies were observational, which introduces potential residual confounding and selection bias despite generally high methodological quality. Second, statistical heterogeneity was substantial (*I*^2^ = 80.32%), likely reflecting variation in patient populations, clinical settings, AKI definitions, sampling times, and KIM-1 assay platforms. Third, differences in diagnostic criteria (nKDIGO, AKIN, nRIFLE) and urine collection timing may reduce comparability across studies. Fourth, many studies enrolled relatively small numbers of AKI cases and were conducted in single-center NICU, limiting precision and generalizability. Finally, inconsistent reporting of thresholds and limited follow-up prevented determination of optimal diagnostic cutoffs or assessment of long-term renal outcomes.

In conclusion, this meta-analysis provides compelling evidence that uKIM-1 levels are significantly elevated in neonates with AKI across various clinical contexts. Despite heterogeneity and methodological differences, the consistency of findings supports its potential role in early detection and management of neonatal AKI. Integrating uKIM-1 measurement into clinical protocols could enhance early diagnosis, trigger timely interventions, and ultimately improve neonatal outcomes. Future studies should aim to standardize biomarker reporting and incorporate uniform AKI definitions to facilitate meta-analytic synthesis and clinical translation. Moreover, longitudinal studies are needed to assess the predictive value of uKIM-1 over time and its relationship with long-term renal outcomes.

## 5. Conclusions

uKIM-1 is significantly elevated in neonates with AKI, supporting its role as a promising non-invasive biomarker for early diagnosis. Despite heterogeneity across studies, the findings were robust and unbiased. To improve comparability and clinical utility, future research should standardize AKI definitions and biomarker protocols. Further longitudinal studies are needed to assess uKIM-1’s predictive value and its integration with multi-marker panels for enhanced diagnostic and prognostic accuracy in neonatal care.

## Figures and Tables

**Table 1:** Characteristics of included studies.

Study	Country (Continent)	Study Design	Gestational Ages (Weeks)	Sampling Time	Conditions	Setting	%Male (AKI, Non-AKI)	AKI Definition	AKI (*n*)	Non-AKI (*n*)	Assay	Value of uKIM-1	Unit
Ahn et al., 2020 [45]	Republic of Korea (Asia)	Prospective cohort	28–32	The first 7 days of life	Premature infants	NICU	50, 55	nKDIGO	4	32	Multiplex Luminex assay^®^ (ELISA)	Mean ± SD	KIM-1/Cr (ng/mg)
Askenazi et al., 2011 [46]	USA (South America)	Nested case–control	<32	The first 6 days of life	Very low birth weight infants	NICU	44.4, 47.6	AKIN	9	21	Prototype duplex (2-plex) assays (ELISA)	Median (IQR)	pg/mL
Askenazi et al., 2012 [47]	USA (South America)	Nested case–control	>34	The first 4 days of life	Birth weight >2000 g	NICU	89, 38	AKIN	9	24	Meso Scale Discovery Human KIM-1 Assay Kit (ELISA)	Mean (95% CI)	ng/mL
Askenazi et al., 2016 [48]	USA (South America)	Prospective cohort	≤31	The first 4 days of life	Preterm infants (BW ≤ 1200 g)	NICU	36, 53	nKDIGO	27	84	Meso Scale Human Kidney Injury Panel 3 Kit Assay (ELISA)	Median (IQR)	pg/mL
Borchet et al., 2021 [49]	Chile (South America)	Descriptive (cohort study)	NR	After induction of anesthesia at 24 h	Neonates < 4 kilograms (kg), with complex congenital heart diseases	NR	67, 50	nRIFLE	9	12	Quantitative immunoassay (ELISA)	Median (IQR)	pg/mL
Elmas et al., 2016 [50]	Turkey (Asia)	Prospective case–control	28–32	The first 7 days of life	Non-septic and non-asphyxiated critically ill neonates	NICU	54, 47	AKIN	13	51	Human KIM-1 ELISA kit	Median (minimum-maximum)	ng/mL
ElSadek et al., 2020 [30]	Egypt (Africa)	Prospective case–control	37–40	3 days after admission	Critically ill neonates	NICU	62, 55	nKDIGO	39	11	Human KIM-1 ELISA kit	Mean ± SD	ng/mL
Genc et al., 2012 [51]	Turkey (Asia)	Prospective cohort	<34	The first 7 days of life	Premature infants with respiratory distress syndrome (RDS)	NICU	50, 66.7	nKDIGO	18	15	ELISA	Mean ± SD	ng/mg creatinine
Mehrkesh et al., 2022 [52]	Iran (Asia)	Case–control	>34	The first 4 days of life	Neonates with asphyxia	NICU	NR	nRIFLE	22	23	ELISA	Mean ± SD	KIM-1 Cr-standardized (ng/mL)
Rumpel et al., 2021 [29]	USA (North America)	Prospective cohort	≥35	The first 3 days of life	Neonates with hypoxic–ischemic encephalopathy receiving therapeutic hypothermia	NICU	63, 44	nKDIGO	16	48	ELISA	Mean ± SD	pg/mL
Sarafidis et al., 2012 [53]	Greece (Europe)	Case–control	≥36	The first 10 days of life	Asphyxiated neonates	NICU	75, 80	nKDIGO	8	5	ELISA	Median (IQR)	pg/mL
Sullenger et al., 2025 [54]	USA (North America)	Prospective cohort	>37	8 to 24 h after separation from bypass	Neonates (≤28 days) undergoing cardiac surgery (CS), late postoperative	NR	NR	nKDIGO	9	4	ELISA	Median (IQR)	pg/mL
Unal et al., 2020 [55]	Turkey (Asia)	Prospective cohort	25–32	The first 2–3 days of life	Very low birth weight preterm infants	NICU	55.6, 63.3	nKDIGO	9	30	ELISA	Mean ± SD	pg/mL

USA, United States of America; AKI, acute kidney injury; KIM-1, kidney injury molecule-1; NICU, neonatal intensive care unit; SD, standard deviation; IQR, interquartile range; ELISA, enzyme-linked immunosorbent assay; AKIN, acute kidney injury network; nKDIGO, modified neonatal kidney disease: improving global outcomes; nRIFLE, neon.

**Table 2:** Newcastle-Ottawa Scale (NOS) quality assessment of included studies evaluating acute kidney injury biomarkers in neonates.

Study	Selection	Comparability	Exposure/Outcome	Total Score (Out of 9)	Quality Classification
Ahn et al. (2020) [45]	4/4	2/2	2/3	8	High
Askenazi et al. (2011) [46]	4/4	2/2	2/3	8	High
Askenazi et al. (2012) [47]	4/4	2/2	2/3	8	High
Askenazi et al. (2016) [48]	4/4	1/2	2/3	7	High
Borchet et al. (2021) [49]	3/4	1/2	2/3	6	Moderate
Elmas et al. (2016) [50]	3/4	2/2	2/3	7	High
ElSadek et al. (2020) [30]	3/4	2/2	2/3	7	High
Genc et al. (2012) [51]	3/4	1/2	2/3	6	Moderate
Mehrkesh et al. (2022) [52]	3/4	2/2	2/3	7	High
Rumpel et al. (2021) [29]	4/4	1/2	3/3	8	High
Sarafidis et al. (2012) [53]	4/4	1/2	3/3	8	High
Sullenger et al. (2025) [54]	4/4	1/2	3/3	8	High
Unal et al. (2020) [55]	4/4	1/2	3/3	8	High

**Table 3:** Subgroup analyses of uKIM-1 levels between neonatal with AKI and without AKI.

Subgroup Analyses	*p*-Value Between AKI vs. Non-AKI	Hedges’s g (95% CI)	*I*^2^ (%)	Number of Studies	References
**Continent (test of group difference, *p*-value < 0.0001)**					
Africa	< 0.0001	2.12 (1.34, 2.90)	N/A	1	[30]
Asia	0.065	0.79 (−0.05, 1.62)	84.81	5	[45,50,51,52,55]
Europe	0.237	0.64 (−0.42, 1.71)	N/A	1	[53]
North America	0.160	0.39 (−0.15, 0.93)	68.17	5	[29,46,47,48,54]
South America	0.290	−0.45 (−1.30, 0.39)	N/A	1	[49]
**Study design (test of group difference, *p*-value = 0.95)**					
Case–control	0.128	0.60 (−0.17, 1.38)	84.84	6	[30,46,47,50,52,53]
Cohort	0.038	0.63 (0.02, 1.23)	78.54	7	[29,45,48,49,51,54,55]
**Sampling time (test of group difference, *p*-value = 0.86)**					
The first 2–4 days of life	0.002	0.76 (0.27, 1.26)	68.21	5	[29,47,48,52,55]
The first 6–10 days of life	0.208	0.54 (−0.30, 1.37)	81.05	5	[45,46,50,51,53]
Others	0.650	0.42 (−1.39, 2.22)	91.73	3	[30,49,54]
**AKI definition (test of group difference, *p*-value = 0.10)**					
AKIN	0.933	−0.03 (−0.74, 0.68)	66.95	3	[46,47,50]
nKDIGO	0.001	0.96 (0.38, 1.54)	79.21	8	[29,30,45,48,51,53,54,55]
nRIFLE	0.699	0.27 (−1.08, 1.61)	85.24	2	[49,52]

Abbreviations: CI, confidence interval; N/A, not assessed nKDIGO, modified neonatal kidney disease: improving global outcomes; AKIN, acute kidney injury network; nRIFLE, neonatal: risk, injury, failure, loss, end-stage kidney disease.

## Data Availability

All data generated or analyzed during this study are included in this published article and its Supplementary Materials.

## Supplementary Materials

The following supporting information can be downloaded at: https://www.mdpi.com/article/10.3390/life15121842/s1, Figure S1: Subgroup analysis of the association between uKIM-1 and AKI in neonates, stratified by continent; Figure S2: Subgroup analysis of the association between uKIM-1 and AKI in neonates, stratified by study design; Figure S3: Subgroup analysis of the association between uKIM-1 and AKI in neonates, stratified by sampling time; Figure S4: Subgroup analysis of the association between uKIM-1 and AKI in neonates, stratified by AKI definition; Table S1: PRISMA 2020 checklist; Table S2: Literature search in PubMed; Table S3: Literature search in Scopus; Table S4: Literature search in Embase; Table S5: Literature search in Web of Science; Table S6: Literature search in Cochrane Library; Table S7: Interrater reliability for title-abstract screening; Table S8: Interrater reliability for full-text screening; Table S9: Newcastle-Ottawa Scale assessment of included studies by domain; Table S10: GRADE summary of findings: uKIM-1 levels for early detection of AKI.

## Abbreviations

The following abbreviations are used in this manuscript:

AKI	Acute kidney injury
AKIN	Acute kidney injury network
ApoM	Apolipoprotein M
CI	Confidence Interval
CS	Cardiac surgery
ELISA	Enzyme-linked immunosorbent assay
GDF-15	Growth differentiation factor 15
IGFBP7	Insulin-like growth factor-binding protein 7
IL-18	Interleukin-18
IQR	Interquartile range
KIM-1	Kidney injury molecule-1
L-FABP	Liver-type fatty acid binding protein
MCP-1	Monocyte chemoattractant protein-1
MMP-7	Matrix metalloproteinase-7
NGAL	Neutrophil gelatinase-associated lipocalin
NICU	Neonatal intensive care unit
nKDIGO	Modified neonatal kidney disease: improving global outcomes
NR	Not reported
nRIFLE	Neonatal: risk, injury, failure, loss of kidney function, and end-stage kidney disease
RDS	Respiratory distress syndrome
uKIM-1	Urinary kidney injury molecule-1
UMOD	Uromodulin
