# Supplementary material for: Urinary KIM-1 for Early Detection of Acute Kidney Injury in Neonates: A Systematic Review and Meta-Analysis
Source: Life (Basel). 2025 Nov 30;15(12):1842. doi: 10.3390/life15121842 (PMC12733929; doi:10.3390/life15121842)
Supplement: Supplementary file 1 [file life-15-01842-s001.zip › life-4003189-supplementary.pdf]

Urinary KIM-1 for Early Detection of Acute Kidney Injury in Neonates: A Systematic Review and Meta-Analysis

Manapat Praditaukrit [1], Moragot Chatatikun [2,3*], Aman Tedasen [2,3], Suntornwit Praditaukrit [4], Sirihatai Konwai [4], Jason C. Huang [5], Wiyada Kwanhian Klangbud [6], and Atthaphong Phongphithakchai [4*]

[1] Division of Neonatology, Department of Pediatrics, Faculty of Medicine, Prince of Songkla University, Songkhla, 90110, Thailand; manapat.p@psu.ac.th
[2] School of Allied Health Sciences, Walailak University, Nakhon Si Thammarat 80160, Thailand; moragot.ch@wu.ac.th; aman.te@wu.ac.th
[3] Research Excellence Center for Innovation and Health Products (RECIHP), Walailak University, Nakhon Si Thammarat 80160, Thailand
[4] Nephrology Unit, Division of Internal Medicine, Faculty of Medicine, Prince of Songkla University, Songkhla 90110, Thailand; frankyy1313@gmail.com; sirihataikonwai@gmail.com; atthaphong.p@psu.ac.th
[5] Department of Biotechnology and Laboratory Science in Medicine, National Yang Ming Chiao Tung University, Taipei 112304, Taiwan; jasonhuang@nycu.edu.tw
[6] Medical Technology Program, Faculty of Science, Nakhon Phanom University, Nakhon Phanom 48000, Thailand; wiydakhanhian@gmail.com

* Correspondence: moragot.ch@wu.ac.th (M.C.); atthaphong.p@psu.ac.th (A.P.)

Supplementary Table S1. PRISMA 2020 Checklist

Section and Topic	Item #	Checklist item	Location where item is reported
**TITLE**			
Title	1	Identify the report as a systematic review.	Page 1
**ABSTRACT**			
Abstract	2	See the PRISMA 2020 for Abstracts checklist.	Page 1
**INTRODUCTION**			
Rationale	3	Describe the rationale for the review in the context of existing knowledge.	Pages 1-2
Objectives	4	Provide an explicit statement of the objective(s) or question(s) the review addresses.	Page 3
**METHODS**			
Eligibility criteria	5	Specify the inclusion and exclusion criteria for the review and how studies were grouped for the syntheses.	Page 3
Information sources	6	Specify all databases, registers, websites, organisations, reference lists and other sources searched or consulted to identify studies. Specify the date when each source was last searched or consulted.	Page 3
Search strategy	7	Present the full search strategies for all databases, registers and websites, including any filters and limits used.	Supplementary Tables S2–S6
Selection process	8	Specify the methods used to decide whether a study met the inclusion criteria of the review, including how many reviewers screened each record and each report retrieved, whether they worked independently, and if applicable, details of automation tools used in the process.	Page 3-4
Data collection process	9	Specify the methods used to collect data from reports, including how many reviewers collected data from each report, whether they worked independently, any processes for obtaining or confirming data from study investigators, and if applicable, details of automation tools used in the process.	Pages 4
Data items	10a	List and define all outcomes for which data were sought. Specify whether all results that were compatible with each outcome domain in each study were sought (e.g. for all measures, time points, analyses), and if not, the methods used to decide which results to collect.	Pages 4-5
	10b	List and define all other variables for which data were sought (e.g. participant and intervention characteristics, funding sources). Describe any assumptions made about any missing or unclear information.	Pages 4-5
Study risk of bias assessment	11	Specify the methods used to assess risk of bias in the included studies, including details of the tool(s) used, how many reviewers assessed each study and whether they worked independently, and if applicable, details of automation tools used in the process.	Page 4
Effect measures	12	Specify for each outcome the effect measure(s) (e.g. risk ratio, mean difference) used in the synthesis or presentation of results.	Page 4
Synthesis methods	13a	Describe the processes used to decide which studies were eligible for each synthesis (e.g. tabulating the study intervention characteristics and comparing against the planned groups for each synthesis (item #5)).	Pages 4-5
	13b	Describe any methods required to prepare the data for presentation or synthesis, such as handling of missing summary statistics, or data conversions.	Pages 4-5

Supplementary Table S1. PRISMA 2020 Checklist

Section and Topic	Item #	Checklist item	Location where item is reported
	13c	Describe any methods used to tabulate or visually display results of individual studies and syntheses.	Pages 4-5
	13d	Describe any methods used to synthesize results and provide a rationale for the choice(s). If meta-analysis was performed, describe the model(s), method(s) to identify the presence and extent of statistical heterogeneity, and software package(s) used.	Pages 4-5
	13e	Describe any methods used to explore possible causes of heterogeneity among study results (e.g. subgroup analysis, meta-regression).	Pages 4-5
	13f	Describe any sensitivity analyses conducted to assess robustness of the synthesized results.	Pages 4-5
Reporting bias assessment	14	Describe any methods used to assess risk of bias due to missing results in a synthesis (arising from reporting biases).	Page 4
Certainty assessment	15	Describe any methods used to assess certainty (or confidence) in the body of evidence for an outcome.	Pages 4
**RESULTS**			
Study selection	16a	Describe the results of the search and selection process, from the number of records identified in the search to the number of studies included in the review, ideally using a flow diagram.	Page 5
	16b	Cite studies that might appear to meet the inclusion criteria, but which were excluded, and explain why they were excluded.	Page 5-6
Study characteristics	17	Cite each included study and present its characteristics.	Pages 6, 8-9
Risk of bias in studies	18	Present assessments of risk of bias for each included study.	Page 7, 10
Results of individual studies	19	For all outcomes, present, for each study: (a) summary statistics for each group (where appropriate) and (b) an effect estimate and its precision (e.g. confidence/credible interval), ideally using structured tables or plots.	Pages 7, 10-13
Results of syntheses	20a	For each synthesis, briefly summarise the characteristics and risk of bias among contributing studies.	Pages 6-10
	20b	Present results of all statistical syntheses conducted. If meta-analysis was done, present for each the summary estimate and its precision (e.g. confidence/credible interval) and measures of statistical heterogeneity. If comparing groups, describe the direction of the effect.	Pages 7, 10-13
	20c	Present results of all investigations of possible causes of heterogeneity among study results.	Pages 7, 10-13
	20d	Present results of all sensitivity analyses conducted to assess the robustness of the synthesized results.	Pages 11-12
Reporting biases	21	Present assessments of risk of bias due to missing results (arising from reporting biases) for each synthesis assessed.	Page 7
Certainty of evidence	22	Present assessments of certainty (or confidence) in the body of evidence for each outcome assessed.	Pages 7

Supplementary Table S1. PRISMA 2020 Checklist

Section and Topic	Item #	Checklist item	Location where item is reported
**DISCUSSION**			
Discussion	23a	Provide a general interpretation of the results in the context of other evidence.	Pages 13-15
	23b	Discuss any limitations of the evidence included in the review.	Pages 13-15
	23c	Discuss any limitations of the review processes used.	Page 15
	23d	Discuss implications of the results for practice, policy, and future research.	Page 14-15
**OTHER INFORMATION**			
Registration and protocol	24a	Provide registration information for the review, including register name and registration number, or state that the review was not registered.	Page 3
	24b	Indicate where the review protocol can be accessed, or state that a protocol was not prepared.	Page 3
	24c	Describe and explain any amendments to information provided at registration or in the protocol.	Not applicable
Support	25	Describe sources of financial or non-financial support for the review, and the role of the funders or sponsors in the review.	Page 17
Competing interests	26	Declare any competing interests of review authors.	Page 18
Availability of data, code and other materials	27	Report which of the following are publicly available and where they can be found: template data collection forms; data extracted from included studies; data used for all analyses; analytic code; any other materials used in the review.	Page 18

Supplementary Table S2. Literature search in PubMed.

Search Number	Search Details	Results
**1**	"neonate"[Title/Abstract]	39,900
**2**	"neonates"[Title/Abstract]	94,435
**3**	"neonatal"[Title/Abstract]	271,983
**4**	"newborn"[Title/Abstract]	168,320
**5**	"newborns"[Title/Abstract]	72,403
**6**	"infant"[Title/Abstract]	272,558
**7**	"infants"[Title/Abstract]	331,132
**8**	"neonate"[Title/Abstract] OR "neonates"[Title/Abstract] OR "neonatal"[Title/Abstract] OR "newborn"[Title/Abstract] OR "newborns"[Title/Abstract] OR "infant"[Title/Abstract] OR "infants"[Title/Abstract]	837,491
**9**	"aki"[Title/Abstract]	28,150
**10**	"acute kidney injury"[Title/Abstract]	48,983
**11**	"renal failure"[Title/Abstract]	101,358
**12**	"renal injury"[Title/Abstract]	15,687
**13**	"acute renal injury"[Title/Abstract]	1,479
**14**	"aki"[Title/Abstract] OR "acute kidney injury"[Title/Abstract] OR "renal failure"[Title/Abstract] OR "renal injury"[Title/Abstract] OR "acute renal injury"[Title/Abstract]	160,433
**15**	"kim-1"[Title/Abstract]	93,851
**16**	"kidney injury molecule 1"[Title/Abstract]	2,282
**17**	"kim-1"[Title/Abstract] OR "kidney injury molecule 1"[Title/Abstract]	3,411
**18**	"HDL"[Title/Abstract]	80,002
**19**	"Lipids"[Title/Abstract]	185,584
**20**	"lipids"[MeSH Terms]	1,314,653
**21**	("neonate"[Title/Abstract] OR "neonates"[Title/Abstract] OR "neonatal"[Title/Abstract] OR "newborn"[Title/Abstract] OR "newborns"[Title/Abstract] OR "infant"[Title/Abstract] OR "infants"[Title/Abstract]) AND ("aki"[Title/Abstract] OR "acute kidney injury"[Title/Abstract] OR "renal failure"[Title/Abstract] OR "renal injury"[Title/Abstract] OR "acute renal injury"[Title/Abstract]) AND ("kim-1"[Title/Abstract] OR "kidney injury molecule 1"[Title/Abstract])	69

Supplementary Table S3. Literature search in Scopus.

Search Number	Search Details	Results
1	TITLE-ABS-KEY (neonate)	153,703
2	TITLE-ABS-KEY (neonates)	153,703
3	TITLE-ABS-KEY (neonatal)	362,708
4	TITLE-ABS-KEY (newborn)	1,047,123
5	TITLE-ABS-KEY (newborns)	1,047,123
6	TITLE-ABS-KEY (infant)	1,590,977
7	TITLE-ABS-KEY (infants)	1,590,977
8	(TITLE-ABS-KEY (neonate)) OR (TITLE-ABS-KEY (neonates)) OR (TITLE-ABS-KEY (neonatal)) OR (TITLE-ABS-KEY (newborn)) OR (TITLE-ABS-KEY (newborns)) OR (TITLE-ABS-KEY (infant)) OR (TITLE-ABS-KEY (infants))	2,001,035
9	TITLE-ABS-KEY (aki)	31,487
10	TITLE-ABS-KEY (acute kidney injury)	90,483
11	TITLE-ABS-KEY (renal failure)	351,716
12	TITLE-ABS-KEY (renal injury)	117,520
13	TITLE-ABS-KEY (acute renal injury)	57,125
14	(TITLE-ABS-KEY (aki)) OR (TITLE-ABS-KEY (acute kidney injury)) OR (TITLE-ABS-KEY (renal failure)) OR (TITLE-ABS-KEY (renal injury)) OR (TITLE-ABS-KEY (acute renal injury))	448,984
15	TITLE-ABS-KEY (kim-1)	3,219
16	TITLE-ABS-KEY (kidney injury molecule-1)	6,260
17	(TITLE-ABS-KEY (kim-1)) OR (TITLE-ABS-KEY (kidney injury molecule-1))	7,395
18	((TITLE-ABS-KEY (neonate)) OR (TITLE-ABS-KEY (neonates)) OR (TITLE-ABS-KEY (neonatal)) OR (TITLE-ABS-KEY (newborn)) OR (TITLE-ABS-KEY (newborns)) OR (TITLE-ABS-KEY (INFANT)) OR (TITLE-ABS-KEY (infant))) AND ((TITLE-ABS-KEY (aki)) OR (TITLE-ABS-KEY (acute kidney injury)) OR (TITLE-ABS-KEY (renal failure)) OR (TITLE-ABS-KEY (renal injury)) OR (TITLE-ABS-KEY (acute renal injury))) AND ((TITLE-ABS-KEY (kim-1)) OR (TITLE-ABS-KEY (kidney injury molecule-1)))	224

Supplementary Table S4. Literature search in Embase.

Search Number	Search Details	Results
**1**	neonate.ab,ti.	45597
**2**	neonates.ab,ti.	130586
**3**	neonatal.ab,ti.	360943
**4**	newborn.ab,ti.	171835
**5**	newborns.ab,ti.	100693
**6**	infant.ab,ti.	251132
**7**	infants.ab,ti.	403433
**8**	1 or 2 or 3 or 4 or 5 or 6 or 7	982993
**9**	aki.ab,ti.	53178
**10**	acute kidney injury.ab,ti.	80938
**11**	renal failure.ab,ti.	149264
**12**	renal injury.ab,ti.	23047
**13**	acute renal injury.ab,ti.	2481
**14**	9 or 10 or 11 or 12 or 13	249369
**15**	kim-1.ab,ti.	4819
**16**	kidney injury molecule-1.ab,ti.	3287
**17**	15 or 16	5799
**18**	8 and 14 and 17	122

Supplementary Table S5. Literature search in Web of Science.

Search Number	Search Details	Results
1	neonate (All Fields)	97176
2	ALL=(neonates)	96615
3	ALL=(neonatal)	267824
4	ALL=(newborn)	157116
5	ALL=(newborns)	144863
6	ALL=(infant)	422248
7	ALL=(infants)	415169
8	#1 OR #2 OR #3 OR #4 OR #5 OR #6 OR #7	45591
9	ALL=(aki)	86010
10	ALL=(acute kidney injury)	73449
11	ALL=(renal failure)	144540
12	ALL=(renal injury)	93327
13	ALL=(acute renal injury)	49297
14	#9 OR #10 OR #11 OR #12 OR #13	254022
15	ALL=(kim-1)	3200
16	ALL=(kidney injury molecule-1)	3855
17	#15 OR #16	4985
18	#8 AND #14 AND #17	115

Supplementary Table S6. Literature search in Cochrane Library.

Search Details	Results
("neonate" OR "neonates" OR "neonatal" OR "newborn" OR "newborns" OR "infant" OR "infants"):ti,ab,kw AND ("aki" OR "acute kidney injury" OR "renal failure" OR "renal injury" OR "acute renal injury"):ti,ab,kw AND ("kim-1" OR "kidney injury molecule-1"):ti,ab,kw	9

Supplementary Table S7. Interrater reliability for title-abstract screening.

Cohen's Kappa (κ = 0.87)	**Rater 2 (M.C.): Exclude**	**Rater 2 (M.C.): Include**	**Total Rater 1 (M.P.)**
**Rater 1 (M.P.) : Exclude**	63	0	63
**Rater 1 (M.P.) : Include**	2	11	13
**Total Rater 2 (M.C.)**	65	11	76 (Over all)

Supplementary Table S8. Interrater reliability for full-text screening.

Cohen's Kappa (κ = 0.90)	**Rater 2 (M.C.): Exclude**	**Rater 2 (M.C.): Include**	**Total Rater 1 (M.P.)**
**Rater 1 (M.P.) : Exclude**	205	10	215
**Rater 1 (M.P.) : Include**	5	71	76
**Total Rater 2 (M.C.)**	210	81	76 (Over all)

Supplementary Table S9. Newcastle-Ottawa Scale assessment of included studies by domain.

Study	Selection				Comparability	Outcome/Exposure			Total Score
	Exposed Cohort/ Adequate Case Definition	Nonexposed Cohort/ Representativeness of The Cases	Ascertainment of Exposure/ Selection of Controls	Outcome of Interest/ Definition of controls		Assessment of Outcome/Exposure	Length of Follow-Up/Same Method for Cases and Controls	Adequacy of Follow-Up/Non-Response Rate	
Ahn et al., 2020 [45]	*	*	*	*	**	*	*	–	8
Askenazi et al., 2011 [46]	*	*	*	*	**	*	*	–	8
Askenazi et al., 2012 [47]	*	*	*	*	**	*	*	–	8
Askenazi et al., 2016 [48]	*	*	*	*	*	*	*	–	7
Borchet et al., 2021 [49]	*	*	*	–	*	*	*	–	6
Elmas et al., 2016 [50]	*	*	*	–	**	*	*	–	7
ElSadek et al., 2020 [30]	*	*	*	–	**	*	*	–	7
Genc et al., 2012 [51]	*	*	*	–	*	*	*	–	6
Mehrkesh et al., 2022 [52]	*	*	*	–	**	*	*	–	7
Rumpel et al., 2021 [29]	*	*	*	*	*	*	*	*	8
Sarafidis et al., 2012 [53]	*	*	*	*	*	*	*	*	8
Sullenger et al., 2025 [54]	*	*	*	*	*	*	*	*	8
Unal et al., 2020 [55]	*	*	*	*	*	*	*	*	8

Double asterisks (**) indicate two points awarded for comparability when studies controlled for key confounders. A dash (–) indicates that the criterion is not applicable to the study design. Total scores range from 0 to 9, with higher scores indicating better methodological quality.*

Supplementary Table S10. GRADE summary of findings: u KIM-1 levels for early detection of AKI.

Certainty Assessment							No. of Participants		Effect		Certainty
**No. of Studies**	**Study Design**	**Risk of Bias**	**Inconsistency**	**Indirectness**	**Imprecision**	**Other Considerations**	**AKI**	**Non-AKI**	**Relative (95% CI)**	**Absolute (95% CI)**	
uKIM-1 levels in neonates with AKI vs. non-AKI neonates											
13	Observational: Cohort/Case-Control	Not serious [a]	Serious [b]	Not serious [c]	Serious [d]	Not Serious [e]	192	360	Hedges's g 0.62 (0.16–1.07)	-	⊕⊕◯◯ Low

Explanations

a. Risk of bias: Majority of studies rated high quality (NOS 7–8 stars); no downgrade applied.
b. Inconsistency: Substantial heterogeneity across studies (I^2 = 80.32%) despite subgroup analysis; downgraded by two level.
c. Indirectness: All studies focused on neonates and urinary KIM-1 for AKI detection; no downgrade applied.
d. Imprecision: Effect estimate had a wide confidence interval (Hedges's g = 0.62; 95% CI: 0.16–1.07) and relatively small sample size; downgraded by one level.
e. Publication bias: Funnel plot symmetrical; Egger's and Begg's tests non-significant; no downgrade applied.

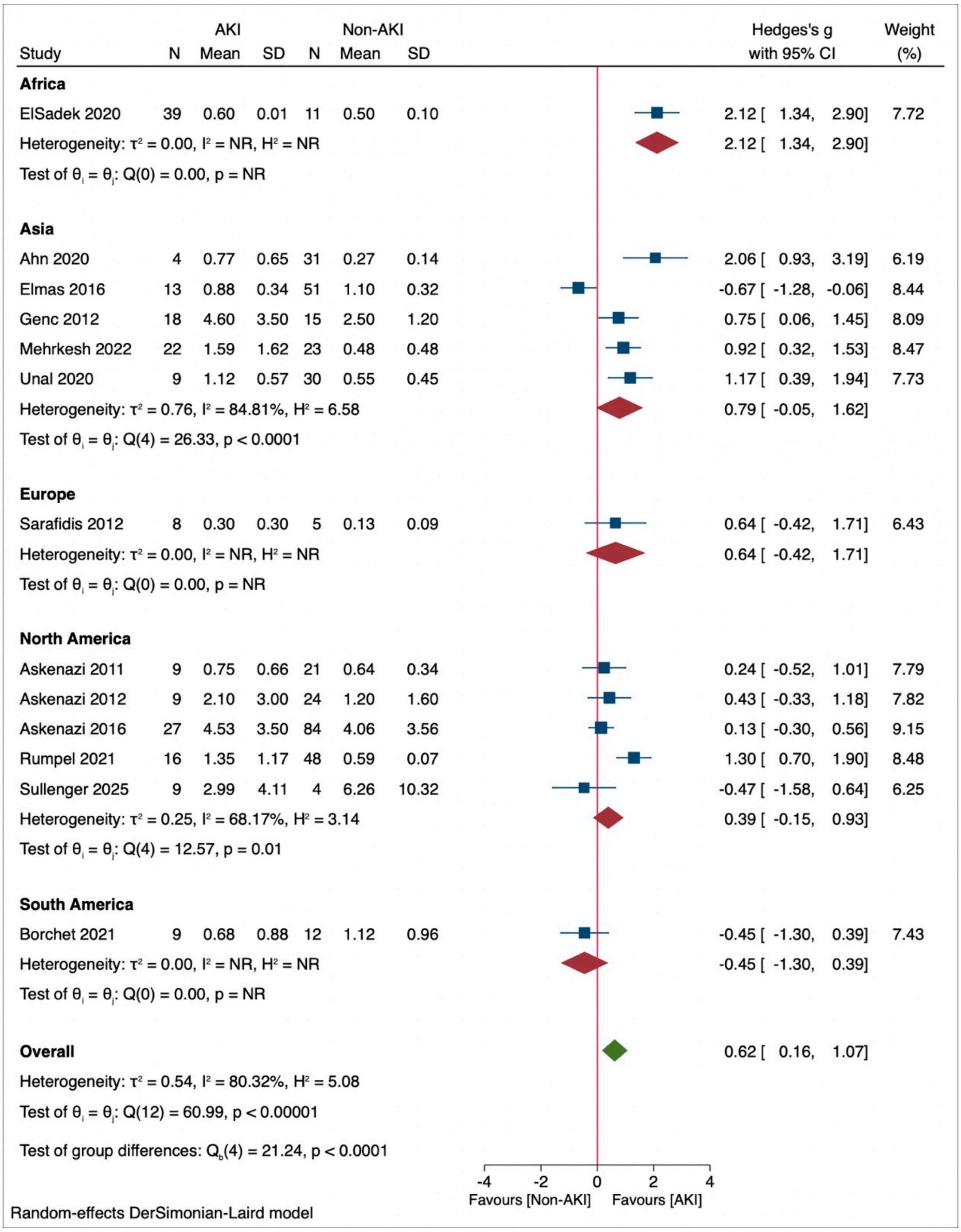

Supplementary Figure S1: Subgroup analysis of the association between uKIM-1 and AKI in neonates, stratified by continent [29-30, 45-55].

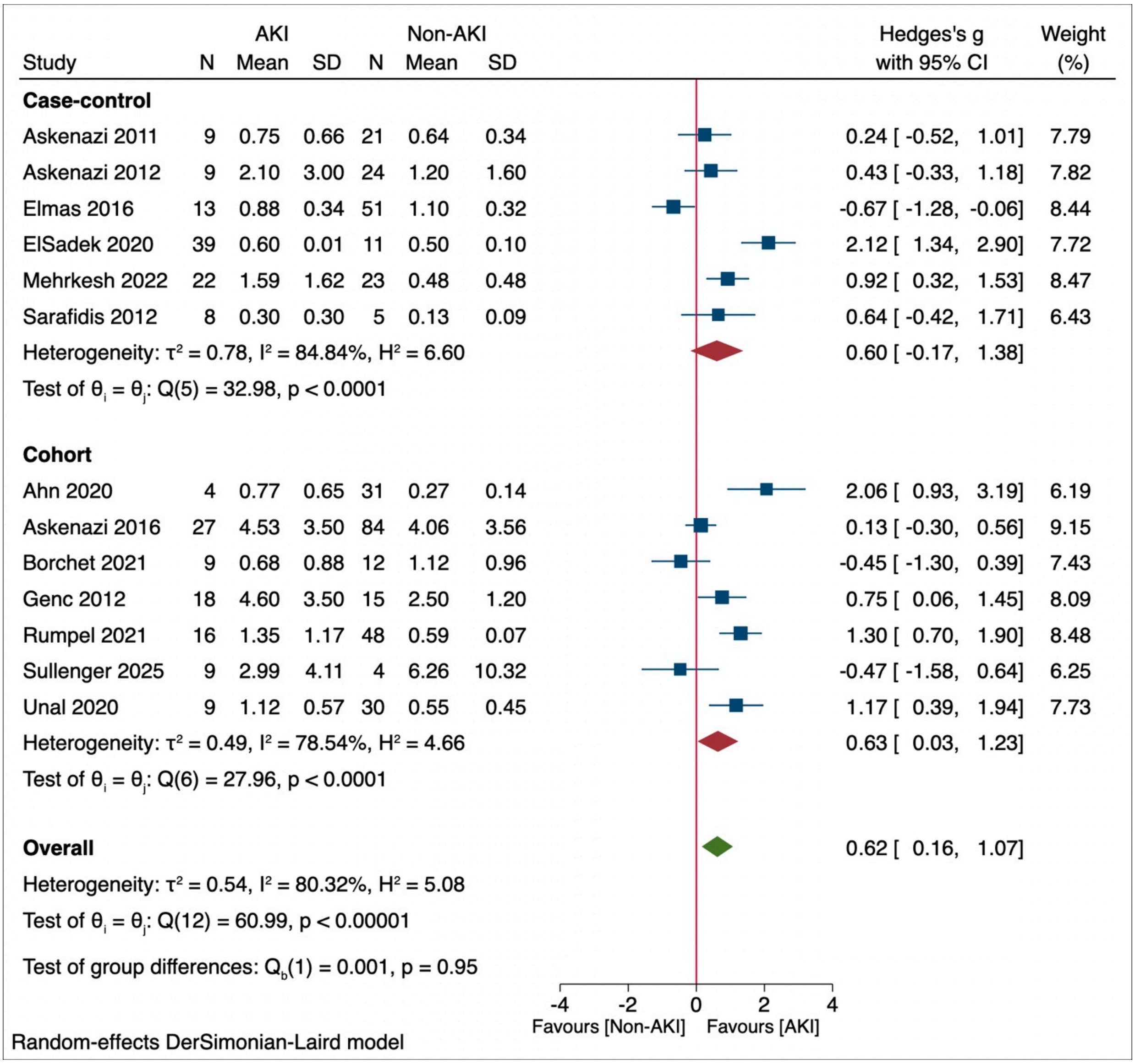

Supplementary Figure S2: Subgroup analysis of the association between uKIM-1 and AKI in neonates, stratified by study design [29-30, 45-55].

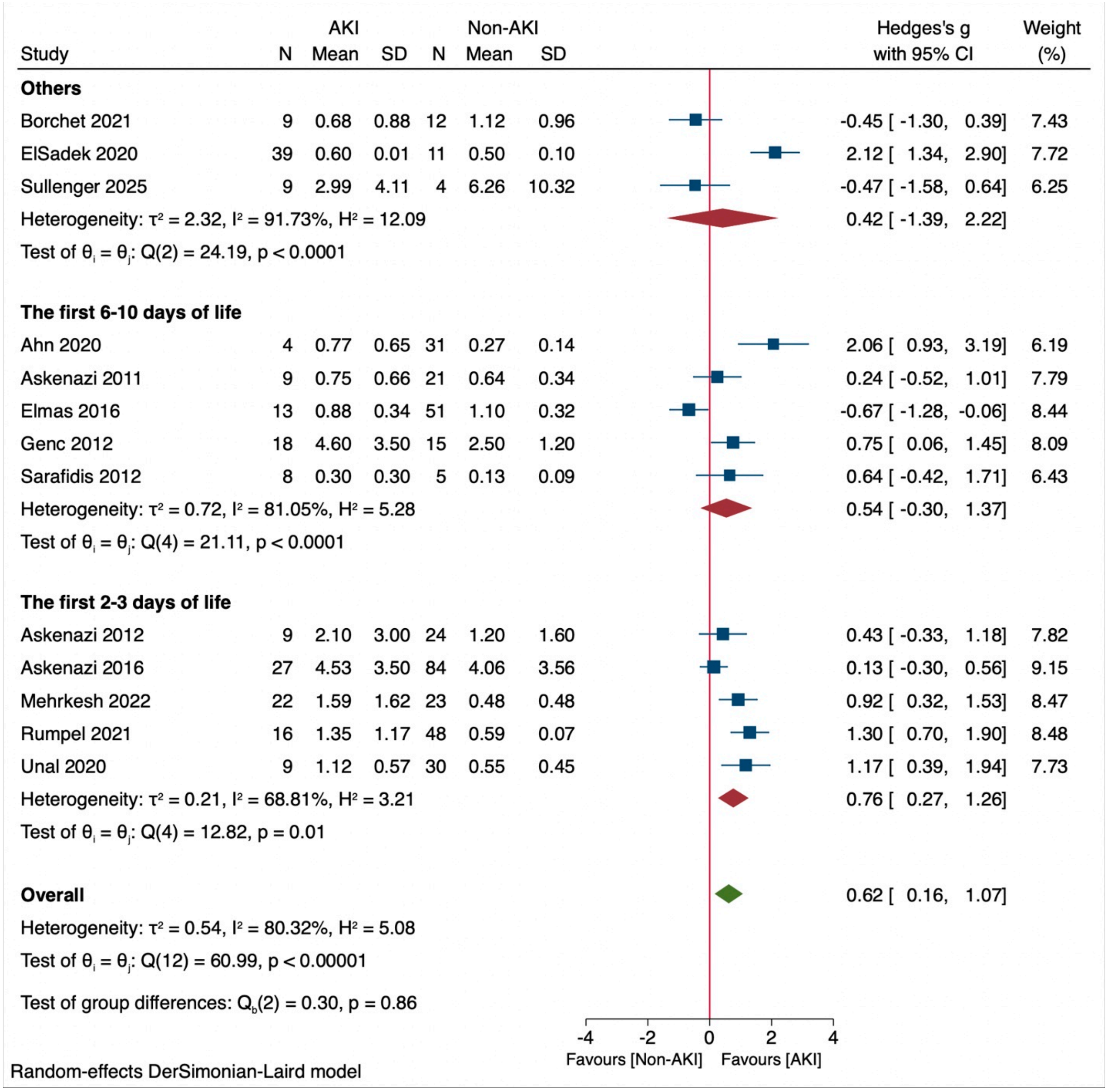

Supplementary Figure S3. Subgroup analysis of the association between uKIM-1 and AKI in neonates, stratified by sampling time [29-30, 45-55].

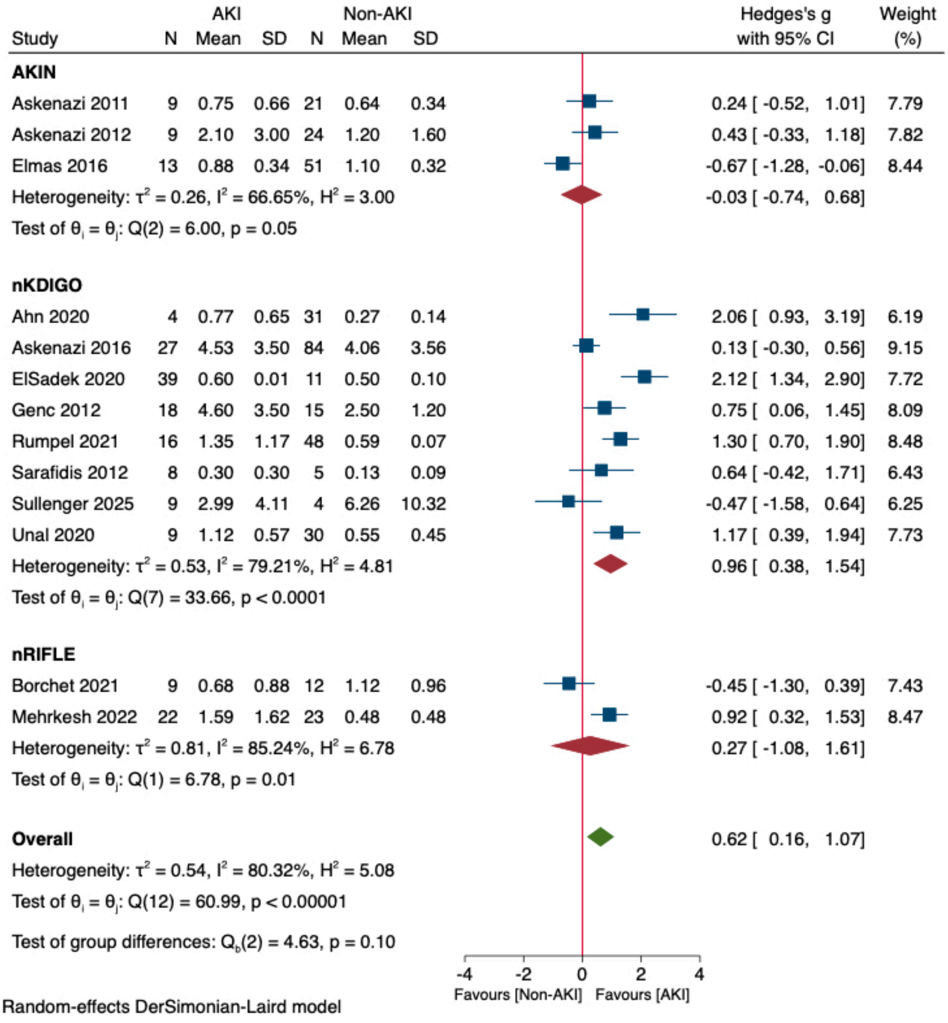

Supplementary Figure S4. Subgroup analysis of the association between urinary KIM-1 and AKI in neonates, stratified by AKI definition [29-30, 45-55].
